# Maternal separation and its developmental consequences on anxiety and parvalbumin interneurons in the amygdala

**DOI:** 10.1007/s00702-023-02657-y

**Published:** 2023-06-09

**Authors:** Mate Abraham, Kirsten Schmerder, Malin Hedtstück, Kimberly Bösing, Annakarina Mundorf, Nadja Freund

**Affiliations:** 1 Division of Experimental and Molecular Psychiatry, Department of Psychiatry, Psychotherapy and Preventive Medicine, LWL University Hospital, Ruhr-University Bochum, Universitätsstraße 150, 44780 Bochum, Germany; 2 Institute for Systems Medicine and Department of Human Medicine, MSH Medical School Hamburg, Hamburg, Germany

**Keywords:** Anxiety, Amygdala, Parvalbumin, Development, Early life stress, Immunohistochemistry

## Abstract

The early postnatal period represents an exceptionally vulnerable phase for the development of neurobiological alterations, aberrant behavior, and psychiatric disorders. Altered GABAergic activity in the hippocampus and the amygdala have been identified in humans diagnosed with depression or anxiety disorders, as well as in respective animal models. Changes in GABAergic activity can be visualized by immunohistochemical staining of parvalbumin (PV) protein. Therewith, alterations in PV intensity as well as in the integrity of the perineural net surrounding PV positive (PV+) interneurons have been reported as consequences of early stress. In the current study, maternal separation (MS) was used to induce early life stress. Female and male Sprague–Dawley rats were subjected to MS over 4 h from postnatal days 2–20. Then, anxiety behavior and PV+ interneurons in the amygdala were analyzed using immunohistochemistry in adolescence or adulthood. MS induced increased anxiety behavior in the marble-burying test in adolescence as well as in the elevated plus maze in adulthood. No effect of sex was found. Concerning alterations of parvalbumin expression in the amygdala, a trend towards a lower number of parvalbumin-positive inhibitory interneurons was shown in the amygdala after MS in adolescence, with no differences in the total number of cells. The current study offers a developmental perspective, suggesting that the kind of anxiety behavior expressed by rats following MS changes over time from active to passive avoidance, indicating that effects of MS are highly dependent on developmental state. Moreover, a cell-type-specific effect of MS on the cellular composition of the amygdala is discussed. The presented study demonstrates the long-lasting consequences of early stress on behavior, offers a possible neurobiological correlate, and discusses possible mediators in the development of these alterations.

## Introduction

Early life stress represents a relevant risk factor for the development of psychopathologies and psychiatric disorders (Walker et al. 2017). The consequences of early adversities have been extensively studied in psychiatric patients, as well as in animal models of psychiatric disorders concerning the alterations they cause. Translational studies demonstrated effects both on a behavioral, and on a cellular level. (Walker et al. 2017; Houwing et al. 2017). A well-established animal model to induce early life stress is maternal separation (MS), which describes the separation of offspring from the dam during early life (Nishi et al. 2013). MS leads to durable behavioral and neurobiological alterations in rodents, similar to changes in humans following adversities in early life (Nishi et al. 2013). Specifically, MS has been shown to increase depressive-like behavior, as well as to induce learned helplessness and working memory deficits (Lukkes et al. 2017; Leussis et al. 2012).

In the past, numerous methods have emerged to model anxiety-like behavior in animals. Marble burying (MB) and elevated plus maze (EPM) have manifested themselves as established methods to assess anxiety in rodents (Jimenez-Gomez et al. 2011; Njung'e and Handley 1991; Bölükbas et al. 2020). MB makes use of the natural rodent behavior of burying novel objects that pose a potential threat (de Boer and Koolhaas 2003). The test is widely used to assess active avoidance, as well as compulsive-like behavior in animal models of psychopathologies (de Boer and Koolhaas 2003; Brouwer et al. 2019). The relevance of MB concerning active fear avoidance has been supported by studies reporting that the application of therapeutic doses of anxiolytic agents could inhibit marble burying while not affecting locomotor activity (Njung'e and Handley 1991). EPM, on the other hand, represents one of the most robust and well-established tests used to assess anxiety, focusing on passive fear avoidance (Korte and de Boer 2003; Carobrez and Bertoglio 2005). The test originates in the conflicting instincts of rodents between exploring novel terrain, and avoiding open spaces (Carobrez and Bertoglio 2005; Rodgers and Dalvi 1997). Multiple studies have reported findings of increased anxiety after MS in multiple behavioral tests, among which were MB and EPM (Troakes and Ingram 2009; Daniels et al. 2004; Lee et al. 2007; Ellis and Honeycutt 2021).

While there is widespread consensus concerning the fact that MS leads to anxiety- and depressive-like behavioral alterations, most studies have focused on outcomes in adulthood (Nishi et al. 2014; Cui et al. 2020; Leussis et al. 2012; Réus et al. 2011). The developmental stage of adolescence has received considerably less attention, even though this crucial time frame might allow for early interventions before the emergence of psychological disorders (Graber 2013). In the current study, two groups of rats that underwent the same MS protocol were subjected to behavioral tests and sacrificed at two different time points: one group in adolescence (P41–42) and one group in adulthood (P61–62).

Concerning neurobiological alterations following early life stress, numerous structures have been proposed to play a role in mediating effects on behavior. The amygdala has been repeatedly shown to be a structure playing a pivotal function in emotional regulation, with alterations identified in a multitude of psychopathologies, especially in fear and avoidance behavior, as well as anxiety disorders (Davis 1992; Rauch et al. 2003; Ocklenburg et al. 2022). The amygdala is integrated into a myriad of neural circuits, with strong reciprocal connections to the ventromedial prefrontal cortex, as well as the hippocampus (Banks et al. 2007). The activity of the amygdala is strongly modulated by inhibitory interneurons utilizing Gamma-Amino Butyric Acid (GABA) as their neurotransmitter (Gildawie et al. 2020). Aberrations in the number and density of these interneurons is a process that has been widely discussed as a possible mediator between early life stress and the development of anxiety-like behavior (Manzano Nieves et al. 2020). GABA is generally considered to have inhibitory activity, leading to post-synaptic hyperpolarization via directly activating Cl^−^ ionophore channels by binding to GABA-A receptors (Bormann 2000). A reduction in GABAergic transmission has therefore been hypothesized to lead to a neurotransmitter disbalance, causing overt excitatory signaling in affected brain regions (Fogaça and Duman 2019). Aberrant GABAergic activity in the hippocampus and the amygdala has been identified in humans suffering from major depressive disorder and anxiety disorders, as well as in animal models of depression and anxiety (Fogaça and Duman 2019).

Parvalbumin (PV) is considered a suitable marker for the immunohistochemical staining of the largest subset of inhibitory interneurons utilizing GABA as their neurotransmitter (Khundakar et al. 2011). The calcium-binding protein PV has been shown to occur colocalized with GABA and to be mostly present in fast-firing Golgi type II interneurons in multiple brain regions (Celio 1990). In the amygdala specifically, interneurons expressing PV immunoreactivity have been identified to exert a regulatory function over excitatory glutamatergic pyramidal neurons, further underlining the role of parvalbumin positive (PV+) neurons in contributing to the regulation of inhibitory/excitatory balance and network synchronization (Khundakar et al. 2011). PV+-interneurons have been demonstrated to play an important role in local circuits in the amygdala: in the basolateral amygdala, the subsection of the amygdala where afferent projections from the prefrontal cortex, the thalamus and the hippocampus terminate PV-Interneurons and glutamatergic principal neurons form local circuits, with interneuron-interneuron and interneuron-principal neuron pairs, thus greatly influencing the intrinsic circuitry of the amygdala (Woodruff and Sah 2007). Alterations in PV intensity (Soares et al. 2020) as well as in the integrity of the perineural net surrounding PV+ interneurons (Gildawie et al. 2020) have been demonstrated as consequences of MS, thus further highlighting the significance of inhibitory PV+ interneurons as part of the neurobiological alterations following MS. Furthermore, inhibition of the activity of PV+ interneurons in the amygdala has been demonstrated to lead to increased anxiogenic behavior (Luo et al. 2020). Moreover, PV+-interneurons in the amygdala have proven to be especially susceptible to early life stress in rodents, as recently reviewed by Ellis and Honeycutt (Ellis and Honeycutt 2021). These findings highlight the role of PV+-interneurons in the amygdala and point to a possible neurobiological link between early life stress and anxiety behavior.

Concerning GABA, the neurotransmitter emitted by PV+ interneurons, numerous findings have suggested an important role in the development of neurological, as well as psychiatric disorders. The translational relevance of these findings is highlighted by alterations in GABAergic signaling that have been demonstrated in patients diagnosed with psychiatric disorders. In suicide victims, Poulter et al. identified a significant decrease in the mRNA expression of the A subunit of the GABA receptor in the hippocampus and the amygdala, the main target of GABA on the postsynaptic membrane (Poulter et al. 2010). Moreover, 5-HT3 agonists have been shown to increase the GABAergic activity of interneurons in the hippocampus as well as the amygdala of depressed patients, thus representing a potential pharmacological mechanism of antidepressants (Gellman and Aghajanian 1993). Furthermore, post-mortem studies in humans have found that transcripts of genes coding for peptides expressed specifically in GABAergic interneurons were downregulated in the amygdala (Guilloux et al. 2012). Following these findings, animal models of depression have confirmed altered GABAergic neurotransmission in the hippocampus and the amygdala: MS has been shown to result in a significant decrease of GABA-A receptors in both of these regions in adult rats (León Rodríguez and Dueñas 2013).

The current study aims to identify alterations in anxiety following MS, as well as alterations in PV density at different developmental stages, thus contributing to a better understanding of the pathomechanisms underlying the long-lasting consequences of early life stress.

## Methods

### Animals

A total of 32 pregnant Sprague–Dawley Rats (Charles River Laboratories, Sulzfeld, Germany) between gestational days 13–15 upon arrival were acquired. Animals were single housed in common Makrolon IV cages under standard conditions (12 h/12 h light/dark period, 22 ± 2 °C room temperature, and 55 ± 25% humidity) with access to food and water ad libitum. Pregnant rats were assigned to either MS or control group (CG) by an independent experimenter. Concerning the offspring, the day of birth was considered postnatal day (P) 0. At P2, all pups were sexed and, when more than ten pups were born in a litter, the litter was culled to a total of ten pups (if possible five males and five females). MS took place on P2-20. Offspring were weaned at P21 and group-housed with same-sex littermates (in groups of two for males and groups of four for females) until being sacrificed. For the present study a total of 19 adolescents (9 females, 10 males) and 25 adults (11 females, 14 males) out of these litters was used. To avoid litter-effects, no more than two animals per litter and sex were selected.

All experiments were conducted in accordance with the German Animal Welfare Act and approved by the ´Landesamt für Natur, Umwelt und Verbraucherschutz Nordrhein-Westfalen´ (LANUV).

### Maternal separation

Pups were separated from the dam and littermates for 4 h daily over P2-20 during the dark (= active) phase as previously described (Mundorf et al. 2020). Until P10, pups were placed on a heating mat adjusted to 37 °C, separated from other littermates using a plastic grid with home cage bedding that allowed auditory and olfactory, but no physical contact. Later, pups were placed in separate cages for MS.

### Behavioral testing

**Fig. 1:**
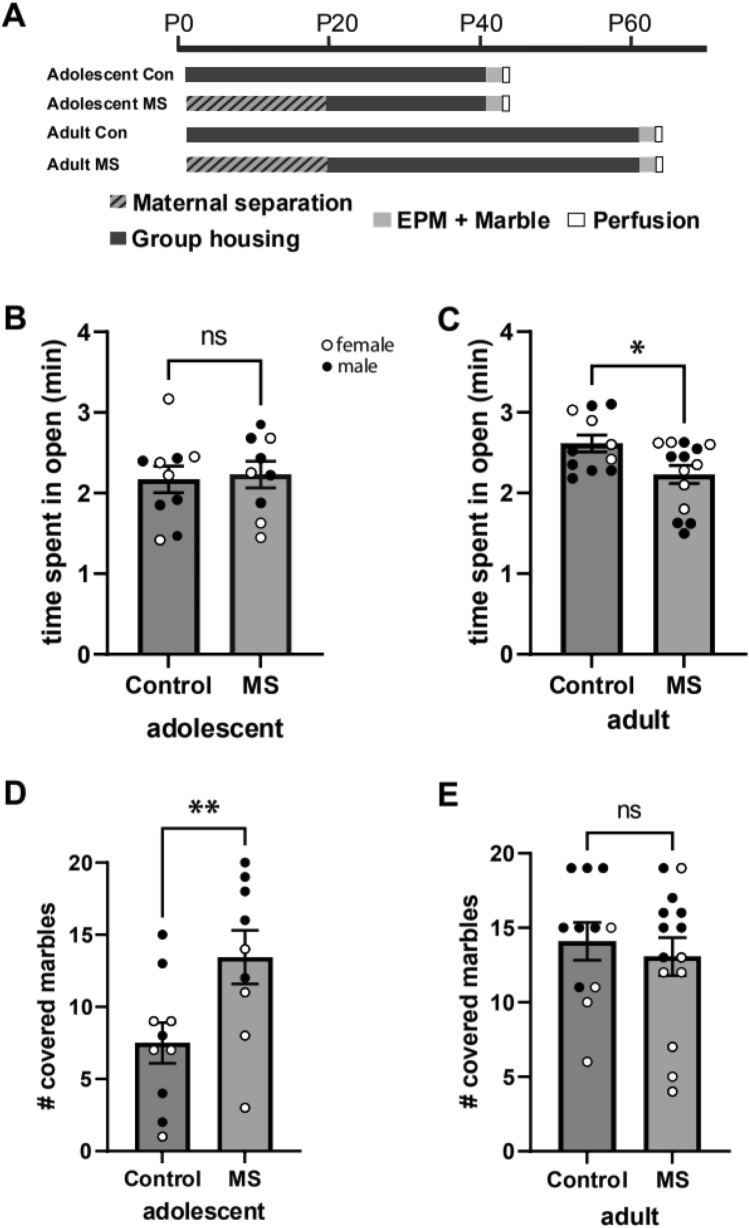
Anxiety behavior in adolescence and adulthood. **A** schematics of the experimental design. The average (+ SEM) time spent in the open arms (minutes) in the EPM is given in adolescence (**B**) and adulthood (**C**). The average (+SEM) number of marbles buried in the MB is given in adolescence (**D**) and adulthood (**E**). MS resulted in significantly less time spent in the open arm in adults but did not affect EPM behavior in adolescence. Whereas MS led to significantly increased marble burying in adolescents but not in adults. Dots represent individual animals with males and females indicated by different colors. *p < 0.05, **p < 0.002

Anxiety-like behavior was assessed in adolescence (P41–42) and adulthood (P61–62) using the elevated plus maze (EPM) followed by the marble burying (MB) test, according to standard protocol utilized by the research group, as described elsewhere (Bölükbas et al. 2020). In brief, the EPM consisted of a plus-shaped maze with two open arms and two closed arms (both 50 cm × 10 cm) at a height of approximately 50 cm. Time spent in the open arms during the five-minute trial time was measured. For MB, 20 marbles in four rows of five marbles each were placed symmetrically in a standard Macrolon IV cage on smoothened bedding. The number of buried marbles after 15 min was counted. Both behavioral tests were carried out in a dark room, with illumination from commercially available LED strips of red light. The experimental design is shown in Fig. 1A.

### Perfusion

One day after behavioral testing, animals were deeply anesthetized with an intraperitoneal ketamine (100 mg/ml; cp-pharma) and xylazine (20 mg/ml; cp-pharma) (ratio 2:1) injection, and then perfused using phosphate buffered saline (0.9%) and paraformaldehyde solution (4%). Brains were then extracted and placed in 4% paraformaldehyde solution for 4 h at 4 °C for postfixation. Afterward, brains were dehydrated using a 30% sucrose solution and stored in an ethylene glycol-based anti-freeze solution at − 20 °C until further use.

### Cryosectioning and immunohistochemistry

Cryosectioning was conducted using the Microme HM 550 (Thermo Fisher Scientific, Massachusetts, USA) at − 20 °C. 40 µm thick coronal sections of the brain at a 10° tilt were obtained. Slices containing the amygdala with the two hemispheres connected by the corpus callosum were identified using a rat brain atlas (Paxinos and Watson 2017). Three slices, containing a total of six individual hemispheres per animal between the bregma positions − 1.92 and − 3.12 were randomly selected for immunochemical staining. The selected slices were stained according to standard free-floating immunofluorescence protocols (Bachman 2013). In brief, slices were blocked for 1 h with 10% normal goat serum (NGS) in 0.3% PBS-T (Triton-X). Then, slices were incubated overnight at 4 °C with Anti-Parvalbumin antibodies from mouse (1:3000, Sigma-Aldrich, P3088) as well as 5% NGS and 0.3% PBS-T. After 24 h, slices were incubated with 0.3% Tween and Anti-Mouse antibodies (1:300, Invitrogen, Alexa Fluor 488, A21202) as well as 10% NGS and 0.3% PBS-T at room temperature. In addition, 4′,6-diamidin-2-phenylindol (DAPI) was utilized to stain all nuclei. Finally, slices were mounted on standard microscope slides (Fisherbrand, Thermo Fisher Scientific, Waltham, Massachusetts, USA).

### Microscopy and cell counting

Images of the amygdala from stained sections were obtained using a fluorescence microscope (Axio Imager M1, Zeiss, Oberkochen, Germany) and utilizing the program “Zen”, version 2.3 (Zeiss, Oberkochen, Germany). The amygdala was defined according to Paxinos and Watson (Paxinos and Watson 2017). After image acquisition, automated analysis of the anatomical regions was carried out using the NIH Program FIJI, Version 2.9.0. The algorithm “Yen” was utilized to define threshold values in the PV and DAPI channels (Yen et al. 1995). The number of cells expressing DAPI staining, as well as those expressing PV staining was determined. The total number of positive cells was calculated to determine the values for each animal.

### Statistical analysis

Statistical analysis was conducted with the software SPSS 27 (IBM). Data were analyzed using three-way ANOVA with the factors sex, age, and MS. Student’s t-test served as posthoc analysis to compare between sexes and the MS and control group.

## Results

### Behavioral testing

For time spent in the open arm there were no significant differences between groups but a trend for age (F(1, 35) = 3.24, p = 0.08). Number of buried marbles were influenced by age (F(1, 35) = 5.86, p = 0.021), sex (F(1, 35) = 18.62, p < 0.001) and MS (F(1, 35) = 4.65, p = 0.038) and an interaction of MS and age ((F(1, 35) = 5.58, p = 0.024). Posthoc t-test revealed that males buried more marbles compared to females (male: M = 14.6; SE = 0.9; female: M = 29.1, SE = 1; t(42) = 4, p < 0.001). No significant difference was found in time spent in the open arm of the EPM in adolescence, however in adulthood, the MS group spent significantly less time in the open arms (M = 2.2; SE = 1.1) compared to controls (M = 2.6; SE = 0.1): t(23) = 2.4, p = 0.023) (Fig. 1B and C). For marble burying in adolescence, MS animals buried more marbles (M = 13.4; SE = 1.9) compared to controls (M = 7.5; SE = 0.4): t(17) = -2.6, p = 0.019 (Fig. 1D). In adults, there was no significant difference (Fig. 1E).

### Immunohistochemistry

**Fig. 2:**
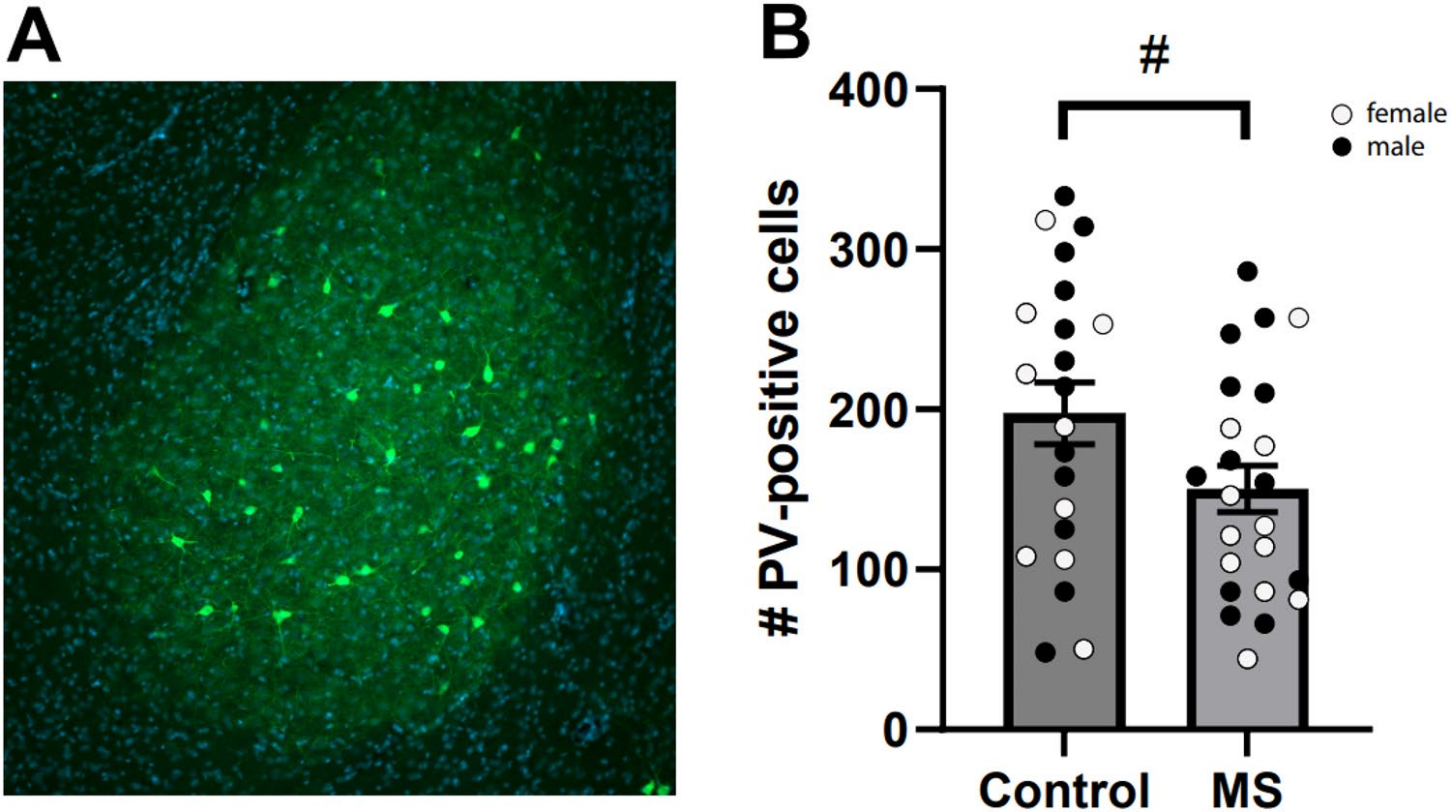
**A** Representative image of immunohistochemical staining of the entire amygdala. **B** The average number of PV+ cells (+ SEM) after MS compared to controls for both ages. Dots represent the mean number of PV+ neurons per animal. Adolescent and adult animals are grouped together into MS or control group. MS led to fewer PV+ neurons compared to controls, indicated by a trend (^#^p = 0.055). Dots represent individual animals with males and females indicated by different colors

The number of PV+-cells was not influenced by age (F(1, 35) = 0.01, p = 0.934) or sex ((F(1, 35) = 0.19, p = 0.192) but showed a trend: F(1, 35) = 3.6, p = 0.066. After MS, the number of PV+ neurons was lower (M = 150.2; SE = 14.5) compared to controls (M = 197.5; SE = 19.4): t(42) = -2.0, p = 0.055 (Fig. 2). While the number of DAPI positive cell was influenced by age: F(1, 35) = 8.4, p = 0.006 and sex F(1, 35) = 5.1, p = 0.03; MS had no influence.

## Discussion

The current study aimed to identify behavioral and neurobiological alterations after MS, as well as to investigate alterations at different stages of development. Sprague–Dawley rats were exposed to MS on P2-21. Afterwards, behavioral tests were conducted on P41 in the adolescence group, and on P61 in the adult group. Following behavioral testing, animals were sacrificed and prepared for neurobiological testing.

This study could demonstrate that MS results in long-lasting behavioral alterations. In adolescence, animals exposed to MS buried significantly more marbles, with no significant difference concerning time spent in the open arms in the EPM. In animals tested on P61, a significantly lower amount of time spent in the open arms in the EPM could be demonstrated in the MS group, while no significant difference could be shown in the marble burying test.

The results of the current study are in line with other publications reporting a higher number of buried marbles following MS in adolescence (Mansouri et al. 2020; Jarrar et al. 2022), as well as with studies showing animals exposed to MS spending less time in the open arms of the EPM in adulthood (Huot et al. 2001; Wang et al. 2020; Koe et al. 2016). In the current study we could show that with the same protocol of maternal separation, one type of anxiety behavior is affected in adulthood and one in adolescence. This finding indicates that effects of MS are highly dependent on developmental state.

While MB is a widely used behavioral test for active avoidance behavior, as well as for repetitive, compulsive-like behavior, EPM is an established behavioral test for exploratory-defensive behavior, as well as for passive avoidance (Brouwer et al. 2019; Taylor et al. 2017; Wang et al. 2020). Results of the current study suggest a phase of increased compulsive-like behavior preceding anxiety in adulthood, a hypothesis that has been proposed as a possible prodromal phase of anxiety disorders (Angoa-Pérez et al. 2013; Dixit et al. 2020).

Concerning the results of the EPM presented in the current study, previous studies have shown decreased time spent in the open arms of the EPM after MS, although results have been inconsistent (Carobrez and Bertoglio 2005; Korte and de Boer 2003; Rodgers and Dalvi 1997; Wang et al. 2020; Taylor et al. 2017; Brouwer et al. 2019). A recent meta-analysis by Wang et al. could identify a slightly increased anxiety-like behavior in EPM in rats having been subjected to MS, however this result only showed moderate effect size (Wang et al. 2020). Moreover, studies showed an asymmetry in the funnel plot, thus possibly suggesting a publication bias (Wang et al. 2020). This shows that while most studies have shown increased anxiety-like behavior in the EPM, further studies are required to shed light upon behavioral alterations following MS. Results of the current study may support the hypothesis that conflicting results could partly be attributed to the numerous different MS protocols utilized by different research groups, as well as differing results being attributable to EPM having been conducted at different developmental stages (Wang et al. 2020; Mundorf et al. 2022). Considering these aspects highlights the difficulty of comparing results with different stages of development examined in independent studies. The current study allows for the analysis of the emergence of psychopathologies by yielding results from different age groups after having used the same MS protocol. Furthermore, this study contributes to furthering our understanding of behavioral alterations following MS, offering the possibility that anxiety-like behavior undergoes a shift during development from active avoidance behavior shortly after MS to increased exploratory-defensive behavior in adulthood.

Of note, a recent review demonstrated that carryover effects are possible when the same animal is tested in more than one behavioral test (Cnops et al. 2022). Thus, it is to mention that results in the MB may have been influenced by the previous EPM testing. However, as behavioral tests were conducted in the same order in both groups, an attribution of the presented results to this effect only seems highly improbable.

Concerning humans, replicable results have suggested that avoidance behavior might represent a prodromal phase of anxiety disorders (Fava et al. 1988, 1992). Thus, active avoidance behavior might represent a translational phenomenon preceding anxiety across different species. This finding demonstrates the potential of early screening methods focusing on avoidance behavior as a possible predictive marker for the development of anxiety disorders (Graber 2013). Further studies in patients expressing obsessive behavior are required to evaluate the preventive and therapeutic potentials of early treatment of anxiety disorders.

The impaired cellular composition of the amygdala might represent one of the neurobiological correlates of the observed anxiety-like behavior. Alterations in the number of PV+  Golgi-Type-II inhibitory interneurons have been repeatedly shown to be present in animals having been subjected to MS (Ellis and Honeycutt 2021). In the current study, when looking at both age groups, the number of PV+-cells in the amygdala showed a trend towards less inhibitory interneurons in the MS group. This is in line with findings described elsewhere in literature, as recently reviewed by Ellis and Honeycutt (Ellis and Honeycutt 2021). Interestingly, so far, others have only investigated PV+-cells after early adversity in the lateral and basolateral amygdala but not in the amygdala as a whole (Ellis and Honeycutt 2021). When taking into account that excitatory and inhibitory neuronal activity has opposite effects on anxiety behavior dependent on the amygdala subregion, e.g., central vs. lateral amygdala (Janak and Tye 2015; Tye et al. 2011) analyzing the total cell number may lead to different results than analyzing the subregions separately. However, optogenetic studies demonstrate that the amygdala circuitry (e.g., basolateral-central projections) need to be considered as critical circuit elements for acute anxiety (Tye et al. 2011). Consequently, it is even more interesting that analyzing the whole amygdala leads to similar results found in only the lateral amygdala.

Just as important is the fact that the nuclei of the amygdaloid complex demonstrate structural and functional asymmetries (Ocklenburg et al. 2022) thus differential effects after exposure are possible. Especially given the important role of hemispheric asymmetries in mental disorders (Mundorf and Ocklenburg 2023; Mundorf et al. 2021). As this study did neither differentiate between the nuclei of the amygdaloid complex nor between the left and right hemispheres, this could be attenuating the found effect. Future studies should focus on investigating different amygdaloid nuclei separately, as well as differentiating between hemispheres, to fully unravel the neurobiological alterations caused by MS.

To summarize, the current study yielded significant results, supporting the role of early life stress in the development of anxiety behavior, as well as the presence of a prodromal period of compulsive-like behavior. Furthermore, it points towards a shift in anxiety-like behavior following MS, suggesting a shift from active to passive avoidance behavior. Moreover, it offers a possible neurobiological correlate in the form of a trend toward less PV+ interneurons in the amygdala following MS. Further studies are required to unravel the exact pathomechanisms underlying anxiety behavior following early life stress.

## Limitations and future outlook

While yielding significant results concerning behavioral tests and thus contributing to the understanding of emergence of anxiety following early life adversities, the current study also bears limitations. The study revealed a trend towards a smaller number of PV+-interneurons following MS when analyzing the whole amygdala. While these results are in line with the literature, this is the first study to analyze the PV+-cell numbers in the amygdala as a whole and not only the lateral or basolateral subregion. Given the opposing effects of excitatory and inhibitory activity, this may have influenced the results. Future studies with a larger sample size, as well as a differentiated analysis of the subnuclei, as well as the left and right hemisphere can be expected to yield further insights into neurobiological alterations during development following MS.

## Data Availability

Data availability statement: Data supporting the findings of the current study are available from the corresponding author, [NF], upon reasonable request.

## Abbreviations

DAPI: 4′,6-Diamidin-2-phenylindol
EPM: Elevated plus maze
GABA: Gamma-amino butyric acid
MB: Marble burying
MS: Maternal separation
P: Postnatal day
PV: Parvalbumin
PV+: Parvalbumin positive
